# Loss of the Fbw7 tumor suppressor rewires cholesterol metabolism in cancer cells leading to activation of the PI3K-AKT signalling axis

**DOI:** 10.3389/fonc.2022.990672

**Published:** 2022-09-13

**Authors:** Maria T. Bengoechea-Alonso, Arwa Aldaalis, Johan Ericsson

**Affiliations:** ^1^ Division of Biological and Biomedical Sciences, College of Health and Life Sciences, Hamad Bin Khalifa University, Doha, Qatar; ^2^ School of Medicine and Medical Science, University College Dublin, Dublin, Ireland

**Keywords:** SREBP, Fbw7, PI3K, AKT, cholesterol, cancer

## Abstract

The sterol regulatory-element binding proteins (SREBPs) are transcription factors controlling cholesterol and fatty acid synthesis and metabolism. There are three SREBP proteins, SREBP1a, SREBP1c and SREBP2, with SREBP1a being the strongest transcription factor. The expression of SREBP1a is restricted to rapidly proliferating cells, including cancer cells. The SREBP proteins are translated as large, inactive precursors bound to the endoplasmic reticulum (ER) membranes. These precursors undergo a two-step cleavage process that releases the amino terminal domains of the proteins, which translocate to the nucleus and function as transcription factors. The nuclear forms of the SREBPs are rapidly degraded by the ubiquitin-proteasome system in a manner dependent on the Fbw7 ubiquitin ligase. Consequently, inactivation of Fbw7 results in the stabilization of active SREBP1 and SREBP2 and enhanced expression of target genes. We report that the inactivation of Fbw7 in cancer cells blocks the proteolytic maturation of SREBP2. The same is true in cells expressing a cancer-specific loss-of-function Fbw7 protein. Interestingly, the activation of SREBP2 is restored in response to cholesterol depletion, suggesting that Fbw7-deficient cells accumulate cholesterol. Importantly, inactivation of SREBP1 in Fbw7-deficient cells also restores the cholesterol-dependent regulation of SREBP2, suggesting that the stabilization of active SREBP1 molecules could be responsible for the blunted activation of SREBP2 in Fbw7-deficient cancer cells. We suggest that this could be an important negative feedback loop in cancer cells with Fbw7 loss-of-function mutations to protect these cells from the accumulation of toxic levels of cholesterol and/or cholesterol metabolites. Surprisingly, we also found that the inactivation of Fbw7 resulted in the activation of AKT. Importantly, the activation of AKT was dependent on SREBP1 and on the accumulation of cholesterol. Thus, we suggest that the loss of Fbw7 rewires lipid metabolism in cancer cells to support cell proliferation and survival.

## Introduction

Lipid metabolism is regulated at the cell, tissue, and organismal level to support the need of lipids for membrane/cell growth, or to provide energy during fasting. Lipid metabolism is controlled on several levels, including at the transcriptional level. The SREBP family of transcription factors control cholesterol synthesis and metabolism and contribute to the control of fatty acid and triglyceride synthesis (1–4). There are three members of the family, SREBP1a, SREBP1c, and SREBP2. Most, if not all, cells express SREBP1c and SREBP2, with SREBP1c mainly regulating fatty acid synthesis while SREBP2 controls cholesterol metabolism (5–7). The expression of SREBP1a is restricted to rapidly proliferating cells, including human cancer cells, and is able to activate all known SREBP target genes (7, 8). Although it is unclear how the expression of SREBP1a is activated in cancer cells, it is thought to be important to support the high demand for *de novo* lipid synthesis in cancer cells (9, 10). The SREBP proteins are synthesized as large precursor proteins that are inserted into the endoplasmic reticulum (ER) membrane. In the ER, the SREBPs interact with a chaperone protein, SREBP cleavage-activating protein (SCAP) (6). SCAP in turn interacts with specific ER resident proteins, either INSIG1 (insulin-induced gene 1) or INSIG2. Both SCAP and the INSIG proteins bind cholesterol or cholesterol metabolites, such as 25-hydroxycholesterol, which enhances their interaction and masks an ER export signal in SCAP (11, 12). Thus, cholesterol anchors the SREBP precursor proteins in the ER, thereby preventing their activation. When the levels of cholesterol in the ER are reduced, it no longer interacts with SCAP/INSIG, the ER export signal in SCAP is exposed, and the SCAP-SREBP complex is transported from the ER to the Golgi. In the Golgi, the SREBP precursor proteins are sequentially cleaved by two separate proteases, generating a cytoplasmic N-terminal fragment and a membrane-associated C-terminal fragment that is still bound to SCAP. In the case of SREBP1c, this maturation process is also induced in response to insulin signaling (13–17). Thus, the SREBPs are activated in response to cholesterol depletion and/or insulin signaling. The liberated N-terminal fragments are translocated to the nucleus, where they interact with target gene promoters and regulate their expression.

The active transcription factors have short half-lives and are rapidly degraded by the ubiquitin-proteasome pathway (18). This process is regulated by the GSK3-mediated phosphorylation of the active transcription factors. GSK3 phosphorylates specific serine/threonine residues in nuclear SREBP1 and SREBP2, thereby creating binding sites for the E3 ubiquitin ligase Fbw7, also known as Fbxw7 (19–21). These residues are not phosphorylated in the precursor proteins, and Fbw7 only targets the active transcription factors for degradation. The Fbw7-dependent degradation of active SREBP proteins is inhibited downstream of insulin signaling as a result of AKT-mediated inactivation of GSK3 (19, 21). In cancer cells, the Fbw7-dependent degradation of nuclear SREBP1 is also inhibited during mitosis (22, 23). This process involves the sequential phosphorylation of SREBP1 by two mitotic kinases, CDK1 and PLK1, which interferes with the interaction between SREBP1 and Fbw7 (24). Inactivation of SREBP1 in cancer cells results in a G1 cell cycle arrest (23). This could be explained, at least in part, by the recent finding that SREBP1 enhances the expression of cyclin D1, thereby promoting the CDK4/6-mediated phosphorylation and inactivation of Rb (25).

Apart from targeting active SREBP molecules for degradation, Fbw7 also targets a number of growth-promoting factors for degradation, including c-Myc, c-Jun, and cyclin E (26–28). In fact, Fbw7 is recognized as a tumor suppressor and loss-of-function mutations in the Fbw7 gene are frequently found in human tumors (26, 28, 29). Thus, the activity of this ubiquitin ligase is an attractive therapeutic target in certain human cancers. Fbw7 is an F-box protein and belongs to the SCF (cullin-Skp1-F-box) family of ubiquitin ligases, and its activity is dependent on cullin 1. Fbw7 is recruited to cullin 1 through interactions with the adaptor protein Skp1 and function as the substrate-recognizing component of the SCF complex. Thus, Fbw7 is responsible for the recognition and recruitment of protein substrates but is not directly involved in the ubiquitination reaction as such. There are three Fbw7 isoforms, Fbw7α, Fbw7β, and Fbw7γ, with the α and γ isoforms localized to the nucleus, while the β isoform is localized to the cytoplasm. Most of the Fbw7 substrates described in the literature are targeted by Fbw7α (26, 29). Little is known about the regulation of Fbw7. However, it has been suggested that its dimerization is required for the recognition and degradation of some of its targets (30), and that the different isoforms could have different targets (31). The stability of Fbw7α is also controlled by ubiquitination and deubiquitination by TRIP12 and USP9X, respectively (32, 33).

The PI3K-AKT-mTOR signaling axis is a major regulator of metabolism, proliferation, and survival downstream of multiple receptor tyrosine kinases, including the EGF and insulin receptors (34–38). Both AKT and mTOR are major signaling hubs and the PI3K-AKT-mTOR signaling pathway is frequently activated in human tumors (34, 39–44). Activation of mTOR complex 1 (mTORC1) downstream of AKT is required for the growth factor-dependent regulation of SREBP1c (10, 45–48). In addition, the AKT-mediated inactivation of GSK3 inhibits the Fbw7-dependent degradation of nuclear SREBP1 and -2 (21, 24). The degradation of many other Fbw7 targets is also dependent on GSK3. Thus, the SREBP pathway and Fbw7 are intimately linked to the PI3K-AKT-mTOR pathway. Furthermore, the induction of SREBP-dependent lipid metabolism in cancer cells is dependent on the PI3K/AKT/mTORC1 signaling pathway (47–52). Importantly, SREBP1 is activated in response to transformation of cells with oncogenic mutants of PI3K and Ras (53). Inactivation of SREBP1 in cancer cells attenuates their proliferation, both *in vitro* and in mouse tumor models (23, 24, 49–51, 54, 55). Apart from supporting enhanced lipid synthesis, SREBP1a also controls the expression of cyclin D1 and the subsequent phosphorylation and inactivation of Rb during G1 (25). Consequently, the SREBP pathway and the pathways it controls have emerged as potential targets for cancer therapeutics (35, 50, 51).

In the current manuscript we demonstrate that inactivation of Fbw7 in cancer cells results in the stabilization and activation of nuclear SREBP1a. As a result, the expression of SREBP target genes is induced and becomes resistant to feedback inhibition. Thus, we propose that the loss of Fbw7 promotes sustained lipid synthesis to support the rapid proliferation of cancer cells. Inactivation of Fbw7 also results in a reduction of the maturation/activation of SREBP2. The maturation of SREBP2 in Fbw7-deficient cells is restored by inactivating SREBP1 or by depleting cells of cholesterol, suggesting that the inactivation of SREBP2 maturation is dependent on SREBP1-mediated accumulation of intracellular cholesterol. We propose that this mechanism could help prevent the accumulation of toxic levels of cholesterol and/or its metabolites in cancer cells. We also report that inactivation of Fbw7 results in the activation of AKT. Again, this effect is reversed following inactivation of SREBP1 or cholesterol depletion, suggesting that the activation of AKT is reliant on SREBP1-dependent cholesterol synthesis. This hypothesis is supported by our observation that the expression of exogenous nuclear SREBP1a enhances the activation of AKT in a cholesterol-dependent manner. Based on the results reported in this study, we propose that the loss of the Fbw7 tumor suppressor and the stabilization of nuclear SREBP1a provides cancer cells with multiple advantages. Firstly, it ensures sustained *de novo* lipid synthesis to support proliferation. Secondly, rewiring of cholesterol metabolism downstream of SREBP1a stabilization results in the activation of AKT, an important regulator of cell metabolism, proliferation, and survival. Dysregulated regulation of lipid metabolism is a hallmark of both metabolic disease and cancer, and metabolic disease including obesity is a risk factor for several cancers, including liver and colorectal cancer. Future studies are needed to determine if the mechanisms described in the current study could be therapeutic targets in human cancer treatment.

## Materials and methods

### Cell culture and treatments

HepG2 (HB-8065), MCF7 (HTB-22), HCT116 (CCL-247) and HEK293 (CRL-1573) cells were from ATCC. The HCT116 Fbw7 knockout cells and the corresponding cells reconstituted with Fbw7α were provided by Bruce Clurman and have been described previously (31). All cell culture media and reagents were from Gibco. HepG2 cells were grown in MEM media supplemented with 10% FBS, non-essential amino acids, sodium pyruvate, Glutamax, and antibiotic-antimycotic. The other cell lines were grown in DMEM media supplemented with 10% FBS and all the reagents above.

### Lentivirus production and transduction

HEK293 cells were used to produce lentiviruses expressing the active nuclear SREBP isoforms, dominant negative cullins, mutant Fbw7α and shRNAs. Twelve μg of lentiviral DNA constructs were co-transfected with lentivirus packaging mix (Dharmacon, TLP4606) by calcium phosphate precipitation and the cells were kept in the incubator for 48 hours. Afterwards, the media was collected and filtered through 0.45μm syringe filters and the viruses were stored in aliquots at -80°C. Cells were transduced in regular media supplemented with polybrene (8μg/ml). Twenty-four hours later, puromycin was added at 5μg/ml and the selection continued for 3 to 4 days.

### Cell lysis and immunoblotting

Cells were lysed in buffer A (50 mM HEPES (pH 7.2), 150 mM NaCl, 1 mM EDTA, 20 mM NaF, 2 mM sodium orthovanadate, 10 mM β-glycerophosphate, 1% (w/v) Triton X-100, 10% (w/v) glycerol, 1 mM phenylmethylsulfonyl fluoride, 10 mM sodium butyrate, 1% aprotinin, 0.1% SDS, and 0.5% sodium deoxycholate) and cleared by centrifugation. Cell lysates were resolved by SDS-PAGE and transferred to nitrocellulose membranes (Millipore). When monitoring the phosphorylation of AKT and mTOR, each sample was run out on two separate gels, and one was used for detecting phosphorylated proteins, while the other was used for detecting total AKT and mTOR. To ensure that equal amounts of protein were loaded in each well, the levels of β-actin in the samples were estimated by Western blotting.

### Reagents and antibodies

Mouse anti-actin (A5441) and standard chemicals were from Sigma. Rabbit anti-SREBP1 (H-160), and mouse anti-SREBP2 (1C6) were from Santa Cruz Biotechnology. The rabbit anti-AKT (#4691), phospho-AKT (Ser473, #4058), mTOR (#2983), and phospho-mTOR (Ser2448, #2974) were from Cell Signaling Technology, and the Fbw7 antibody (A301-720A) was from Bethyl Laboratories. Horseradish peroxidase-conjugated anti-mouse and anti-rabbit IgG were from Invitrogen.

### Plasmids and DNA transfections

The expression vectors for nuclear SREBP1a, SREBP1c and SREBP2 have been described previously (19, 21, 22, 56) (30, 31, 44, 52). To generate the lentiviral expression vectors for the active SREBPs, the corresponding cDNAs were subcloned into pLKO-puro FLAG SREBP1, a gift from David Sabatini (Addgene plasmid #32017). The lentiviral Fbw7, SREBP1 and SREBP2 shRNA vectors and the lentiviral expression vector for GFP were from VectorBuilder. The lentiviral expression vectors for the dominant negative versions of cullin 1, 2, 3, 4A, 4B and 5 were gifts from Stephen Elledge (Addgene plasmids 41911, 41912, 41913, 41914, 41915 and 41916), and the leniviral expression of mouse Fbw7α (R505C) was a gift from Rizwan Haq (Addgene plasmid #160104). The LDL receptor and HMG-CoA synthase promoter-reporter genes have been described previously (20, 22, 56). Transfections were performed by calcium phosphate precipitation.

### LipidTox staining of neutral lipid

LipidTOX Green was used according to the manufacturer’s instructions (ThermoFisher). Briefly, cells grown in 12-well plates were fixed in 3.5% (v/v) formaldehyde and washed extensively with PBS. The stain was used at a 1:1000 dilution in PBS. Cells were stained for 2 hours and kept in PBS at 4 °C until imaging. At least 5 random fields/well were captured on an inverted microscope (Olympus IX73) using identical settings and exposure times. Representative images are displayed in the panels. The fluorescence in each image was quantified in ImageJ and corrected for cell numbers. The mean fluorescence intensities -/+ SD across all images within each experimental group are provided in the figures.

### RNA extraction and qPCR

RNA was extracted using Thermo GeneJet RNA Purification Kit. cDNA was generated using Applied Biosystems High-Capacity cDNA Reverse Transcription Kit. For qPCR, PowerUp SYBR Green Master Mix was used (Applied Biosystems), using GAPDH as a reference. The primers designed to amplify target genes were as follows: GAPDH, forward: CCCTTCATTGACCTCAAC and reverse: TACACTGGAAGATGGTGATGGGATT, the LDL receptor, forward: CAATGTCTCACCAAGCTCTG and reverse: TCTGTCTCGAGGGGTAGCTG, and Fbw7, forward AAAGAGTTGTTAGCGGTTCTC and reverse: CCACATCCATACCATCAAACT.

### Luciferase and β-galactosidase assays

Cells were transiently transfected with the indicated promoter-reporter genes in the absence or presence of the indicated expression vectors and/or shRNA. Luciferase activities were determined in duplicate samples as described by the manufacturer (Promega). Cells were also transfected with the β-galactosidase gene as an internal control for transfection efficiency. Luciferase values (relative light units, RLU) were calculated by dividing the luciferase activity by the β-galactosidase activity. The data represent the average −/+ SD of at least three independent experiments performed in duplicates.

### Data analysis

Statistical data analyses were conducted using the GraphPad Prism 8 software, and paired t-tests were applied to all experiments. The standard deviations (SD) were calculated for experimental replicates and the statistical significance set to P < 0.05.

## Results

### Inactivation of cullin 1 stabilizes nuclear SREBP1 and attenuates the cleavage of SREBP2

**Figure 1:**
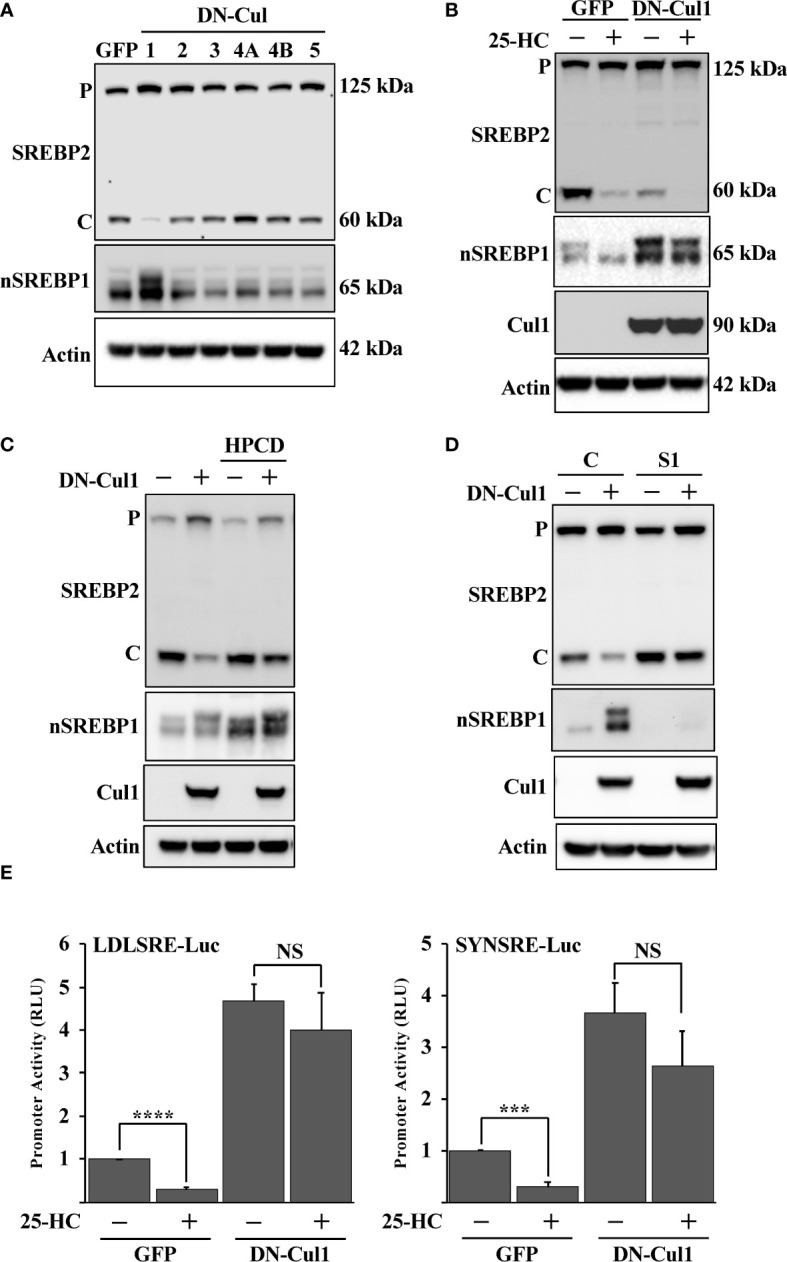
Cullin 1 regulates the maturation of SREBP2. **(A)** HepG2 cells were transduced with lentiviruses expressing the indicated dominant-negative (DN) cullins (cullin 1, 2, 3, 4A, 4B and 5). The levels of SREBP2, nuclear SREBP1 (*nSREBP1*) and β-actin in total lysates were determined by Western blotting. *P* and *C* denotes the precursor and cleaved forms of SREBP2, respectively. **(B)** HepG2 cells were transduced with GFP or DN-cul1 lentiviruses, and left untreated or treated with 25-hydroxycholesterol (25-HC, 1.5 μg/ml) for six hours. The levels of SREBP2, nuclear SREBP1 (*nSREBP1*), cullin 1 (*Cul1*) and β-actin in total lysates were determined by Western blotting. *P* and *C* denotes the precursor and cleaved forms of SREBP2, respectively. **(C)** HepG2 cells were transduced with GFP or DN-cul1 lentiviruses and left untreated or treated with 2-hydroxypropyl-β-cyclodextrin (*HPCD*, 1% (w/v)) for two hours. The levels of SREBP2, nuclear SREBP1 (*nSREBP1*), cullin 1 (*Cul1*) and β-actin in total lysates were determined by Western blotting. *P* and *C* denotes the precursor and cleaved forms of SREBP2, respectively. **(D)** HepG2 cells were transduced with GFP or DN-cul1 lentiviruses, followed by either control (*C*) or SREBP1 (*S1*) shRNA. The levels of SREBP2, nuclear SREBP1 (*nSREBP1*), cullin 1 (*Cul1*) and β-actin in total lysates were determined by Western blotting. *P* and *C* denotes the precursor and cleaved forms of SREBP2, respectively. **(E)** HepG2 cells were transfected with luciferase promoter-reporter constructs containing the SREBP-responsive portions of the human LDL receptor (*LDLSRE-Luc*) and HMG-CoA synthase (*SYNSRE-Luc*) promoters together with empty vector or DN-cul1. Where indicated, the transfected cells were treated with 25-HC for the last 3 hours of the experiment. At the end of the experiment, cells were lysed, and luciferase activity was measured. P-values lower than 0.05 were considered statistically significant. *P < 0.05, **P < 0.01, ***P < 0.001, and ****P < 0.0001. *NS*, not significant.

Fbw7 is the substrate recognizing component of an SCF ubiquitin ligase that targets nuclear SREBP molecules for ubiquitin-mediated degradation. SCF ubiquitin ligases are assembled on scaffold proteins belonging to the cullin family. Fbw7 is recruited to complexes containing cullin 1. In an effort to identify additional SCF complexes regulating the SREBP pathway, HepG2 cells were transduced with lentiviruses expressing dominant-negative (DN) versions of cullin (cul) 1, cul2, cul3, cul4A, cul4B and cul5. As expected, expression of DN-cul1 resulted in the stabilization of nuclear SREBP1 (**Figure 1A**), most likely because the endogenous Fbw7 SCF complex is dependent on cul1 function. Expression of the other DN cullins did not have any significant effect on the levels or activation of SREBP1 or SREBP2. Interesting, the cleavage of SREBP2 was significantly blunted in response to DN-cul1 expression (**Figure 1A**). It is important to point out that the SREBP2 antibody used to monitor SREBP2 cleavage recognizes the C-terminal portion of the protein and not the transcriptionally active N-terminal fragment. The C-terminal portion of SREBP2 remains associated with SCAP in the Golgi and is later transported back to the ER, where it is extracted from the membrane and degraded by the proteasome (57). Since the latter process is independent of Fbw7, we have used the generation of the C-terminal portion to monitor SREBP2 cleavage throughout this study. The cleavage of SREBP2 in control cells expressing GFP was reduced following the addition of 25-hydroxycholesterol (25-HC), a strong inhibitor of SREBP2 cleavage. The cleavage of SREBP2 was already low in cells expressing DN-cul1 resulting in a blunted response to the addition of 25-HC (**Figure 1B**). Interestingly, the cleavage of SREBP2 was restored in DN-cul1 cells treated with 2-hydroxypropyl-β-cyclodextrin (HPCD) (**Figure 1C**), a compound that extracts cholesterol from the plasma membrane, thereby rapidly reducing cellular cholesterol levels. These results suggested that the DN-cul1 cells accumulated excess cholesterol, thereby inhibiting the translocation of the SREBP2-SCAP complex from the ER to the Golgi. Importantly, shRNA-mediated inactivation of SREBP1 also restored the cleavage of SREBP2 in DN-cul1 cells (**Figure 1D**). To test the consequences of cul1 inactivation on the transcriptional level, we used two different SREBP-responsive promoter-reporter genes to monitor SREBP transcriptional activity. Expression of DN-cul1 enhanced the expression of these reporter genes and rendered them insensitive to 25-HC (**Figure 1E**), most likely as a result of the stabilization of nuclear SREBP1. Taken together, these results suggest that the inactivation of cul1 results in the stabilization of nuclear SREBP1 and increased accumulation of cholesterol, thereby preventing the cleavage of SREBP2.

### Inactivation of Fbw7 stabilizes nuclear SREBP1 and attenuates SREBP2 cleavage

**Figure 2:**
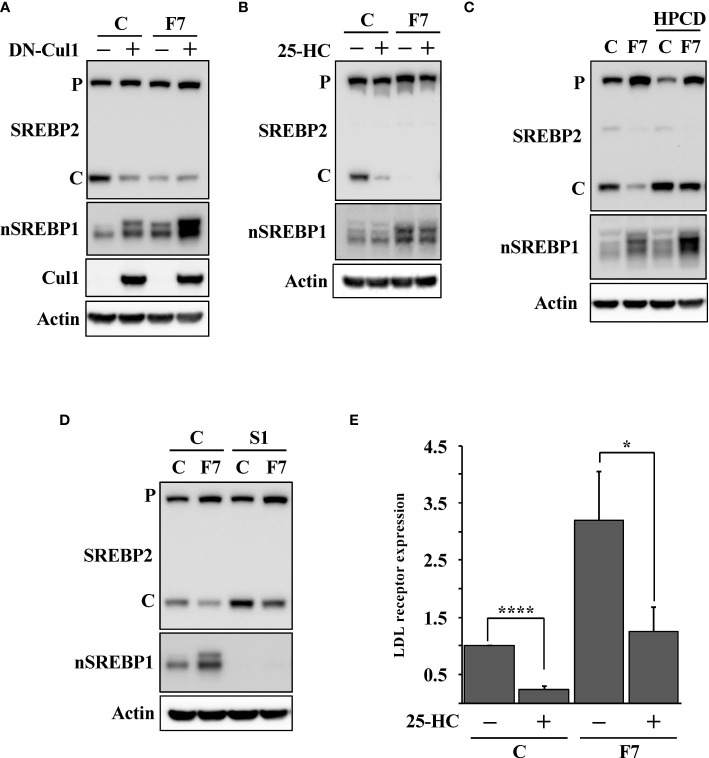
Loss of Fbw7 attenuates the maturation of SREBP2. **(A)** HepG2 cells were transduced with lentiviruses expressing control (*C*) or Fbw7 (*F7*) shRNA, followed by either GFP or DN-cul1. The levels of SREBP2, nuclear SREBP1 (*nSREBP1*), cullin 1 (*Cul1*) and β-actin in total lysates were determined by Western blotting. *P* and *C* denotes the precursor and cleaved forms of SREBP2, respectively. **(B)** HepG2 cells were transduced with control (*C*) or Fbw7 (*F7*) shRNA, and left untreated or treated with 25-HC for six hours. The levels of SREBP2, nuclear SREBP1 (*nSREBP1*) and β-actin in total lysates were determined by Western blotting. *P* and *C* denotes the precursor and cleaved forms of SREBP2, respectively. **(C)** HepG2 cells were transduced with control (*C*) or Fbw7 (*F7*) shRNA and left untreated or treated with HPCD for two hours. The levels of SREBP2, nuclear SREBP1 (*nSREBP1*) and β-actin in total lysates were determined by Western blotting *P* and *C* denotes the precursor and cleaved forms of SREBP2, respectively. **(D)** HepG2 cells were transduced with control (*C*) or Fbw7 (*F7*) shRNA, followed by either control (*C*) or SREBP1 (*S1*) shRNA. The levels of SREBP2, nuclear SREBP1 (*nSREBP1*) and β-actin in total lysates were determined by Western blotting. *P* and *C* denotes the precursor and cleaved forms of SREBP2, respectively. **(E)** HepG2 cells were transduced and treated as in (*B*). The expression of LDL receptor mRNA was determined by real-time qPCR. P-values lower than 0.05 were considered statistically significant. *P < 0.05, **P < 0.01, ***P < 0.001, and ****P < 0.0001. *NS*, not significant.

To test if the expression of DN-cul1 interfered with the function of Fbw7, HepG2 cells were transduced with either control or Fbw7 shRNA, followed by either GFP or DN-cul1. As seen before, expression of DN-cul1 resulted in the stabilization of nuclear SREBP1 and loss of SREBP2 cleavage (**Figure 2A**, compare lanes 1 & 2). The same effect was observed in Fbw7 knockdown cells (**Figure 2A**, compare lanes 1 & 3). As expected, there was no further inhibition of SREBP2 cleavage in Fbw7 knockdown cells in response to DN-cul1 expression (**Figure 2A**, compare lanes 3 & 4), confirming that DN-cul1 attenuates the cleavage of SREBP2 by interfering with Fbw7 function. The cleavage of SREBP2 was very low in Fbw7 knockdown cells and no further reduction was observed in response to 25-HC treatment (**Figure 2B**). However, the cleavage of SREBP2 was restored in response to HPCD-dependent cholesterol depletion (**Figure 2C**), suggesting that the inactivation of Fbw7 results in the accumulation of excess cholesterol, thereby preventing SREBP2 cleavage. Inactivation of SREBP1 partially restored the cleavage of SREBP2 in the Fbw7 knockdown cells (**Figure 2D**), suggesting that the stabilization of nuclear SREBP1 is required to attenuate the cleavage of SREBP2. On the transcriptional level, inactivation of Fbw7 in HepG2 cells activated the expression of the LDL receptor gene and attenuated its sensitivity to 25-HC inhibition (**Figure 2E**). Similar results were obtained using the HMG-CoA synthase promoter-reporter gene (**Figure S1**), i.e., inactivation of Fbw7 reduced the 25-HC sensitivity of the promoter. In agreement with the data presented in **Figure 2D**, inactivation of SREBP1 enhanced the 25-HC sensitivity of the promoter in Fbw7 knockdown cells (**Figure S1**). Thus, our data suggest that the loss of Fbw7 in HepG2 cells results in the stabilization of nuclear SREBP1, resulting in the accumulation of cholesterol and feedback inhibition of SREBP2 maturation. These effects were not limited to HepG2 cells since the inactivation of Fbw7 in MCF7 cells, a human breast cancer cell line, also stabilized nuclear SREBP1 and reduced the cleavage of SREBP2 (**Figure S2**). Importantly, inactivation of SREBP1 in the Fbw7-deficient MCF7 cells restored the maturation of SREBP2 (**Figure S3**).

### Loss of Fbw7α rewires cholesterol metabolism

**Figure 3:**
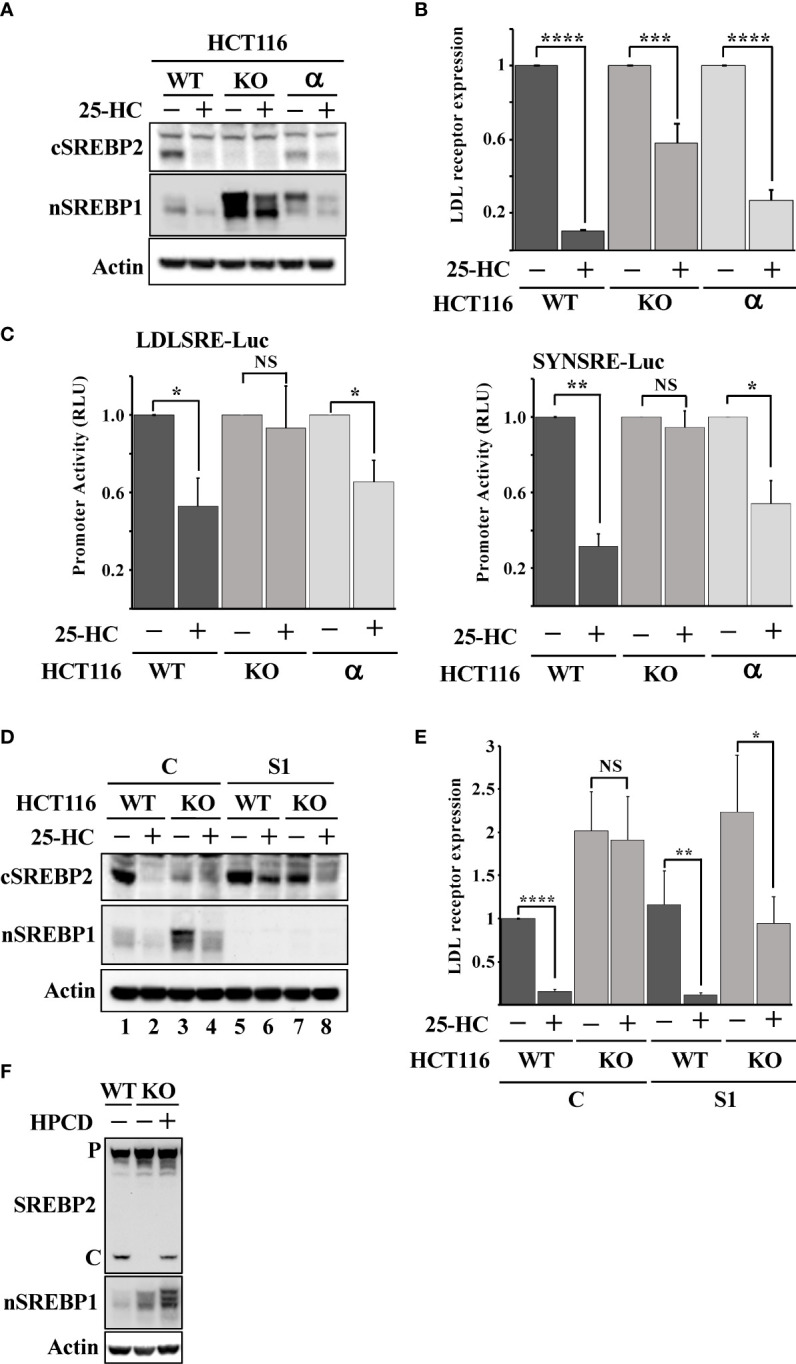
Loss of Fbw7α disrupts cholesterol metabolism. **(A)** HCT116 cells, either wild-type (*WT*), Fbw7 knockout (*KO*), or the same cells reconstituted with Fbw7α (*α*) were treated without **(-)** or with (*+*) 25-HC for 6 hours. The levels of cleaved SREBP2 (*cSREBP2*), nuclear SREBP1 (*nSREBP1*), and β-actin in total lysates were determined by Western blotting. **(B)** HCT116 cells, either wild-type (*WT*), Fbw7 knockout (*KO*), or the same cells reconstituted with Fbw7α (*α*) were treated as in (*A*). The expression of LDL receptor mRNA was determined by real-time qPCR. **(C)** HCT116 cells, either wild-type (*WT*), Fbw7 knockout (*KO*), or the same cells reconstituted with Fbw7α (*α*) were transfected with luciferase promoter-reporter constructs containing the sterol-responsive portions of the human LDL receptor (*LDLSRE-Luc*) and HMG-CoA synthase (*SYNSRE-Luc*) promoters and treated as in (*A*). At the end of the experiment, cells were lysed, and luciferase activity was measured. **(D)** HCT116 cells, either wild-type (*WT*) or Fbw7 knockout (*KO*), were transduced with either control (*C*) or SREBP1 (*S1*) shRNA and left untreated (*-*) or treated (*+*) with 25-HC for 6 hours. The levels of cleaved SREBP2 (*cSREBP2*), nuclear SREBP1 (*nSREBP1*), and β-actin in total lysates were determined by Western blotting. **(E)** HCT116 cells, either wild-type (*WT*) or Fbw7 knockout (*KO*), were transduced and treated as in (*D*). The expression of LDL receptor mRNA was determined by real-time qPCR. **(F)** HCT116 cells, either wild-type (*WT*) or Fbw7 knockout (*KO*), were left untreated (*-*) or treated (*+*) with HPCD for 2 hours. The levels of cleaved SREBP2, nuclear SREBP1 (*nSREBP1*), and β-actin in total lysates were determined by Western blotting. *P* and *C* denotes the precursor and cleaved forms of SREBP2, respectively. P-values lower than 0.05 were considered statistically significant. *P < 0.05, **P < 0.01, ***P < 0.001, and ****P < 0.0001. *NS*, not significant.

There are three isoforms of Fbw7, Fbw7α, Fbw7β and Fbw7γ, and the shRNA-mediated inactivation of Fbw7 results in a partial loss of all isoforms (**Figure S4**). Thus, we took advantage of Fbw7 knockout HCT116 cells to confirm our shRNA results. These cells are deficient of all three Fbw7 isoforms (Fbw7-KO cells). We have previously demonstrated that Fbw7α targets nuclear SREBP1 for degradation (19). To determine if the loss of this same isoform was responsible for the attenuated cleavage of SREBP2, we used Fbw7-KO cells reconstituted with Fbw7α (Fbw7α cells). As expected, the cleavage of SREBP2 was functional and responded to 25-HC in the wild-type cells (**Figure 3A**). However, the Fbw7-KO cells displayed no cleavage of SREBP2 and were, of course, insensitive to 25-HC. Importantly, the cleavage of SREBP2 was restored following reintroduction of Fbw7α and the cleavage responded to 25-HC, suggesting that Fbw7α function is critical to maintain sterol-regulated cleavage of SREBP2 (**Figure 3A**). This notion was supported by the observation that the 25-HC-dependent suppression of the LDL receptor gene was attenuated in the Fbw7-KO cells and partially restored in the Fbw7α cells (**Figure 3B**). Similar results were obtained using two separate SREBP-regulated promoter-reporter constructs in the three HCT116 cell lines (**Figure 3C**). Inactivation of SREBP1 in the Fbw7-KO cells restored SREBP2 cleavage and 25-HC sensitivity (**Figure 3D**, compare lanes 3 & 4 and 7 & 8), suggesting that SREBP1 is required for the defect in SREBP2 maturation observed in Fbw7-KO cells. This notion was supported when this experiment was repeated, and the expression of the LDL receptor gene was analyzed. The expression of LDL receptor mRNA was induced in Fbw7-KO cells and was insensitive to 25-HC suppression (**Figure 3E**). The 25-HC sensitivity of the LDL receptor gene in the Fbw7-KO cells was partially restored following SREBP1 inactivation. HPCD-mediated cholesterol depletion partially restored SREBP2 cleavage in the Fbw7-KO cells (**Figure 3F**), suggesting that the accumulation of cholesterol in these cells is responsible for the attenuated cleavage of SREBP2.

### Fbw7 controls the accumulation of neutral lipids

**Figure 4:**
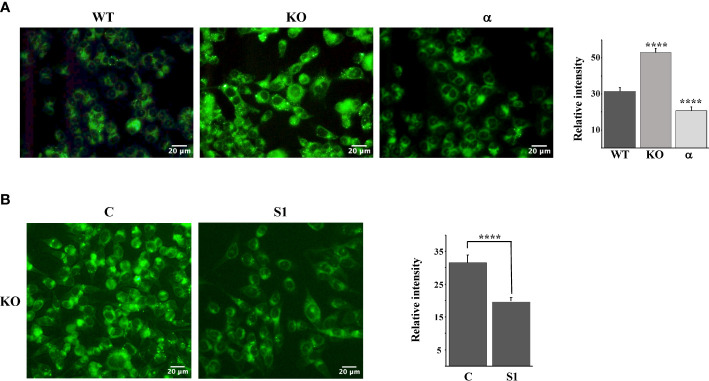
Fbw7 controls the accumulation of neutral lipids. **(A)** HCT116 cells, either wild-type (*WT*), Fbw7 knockout (*KO*), or the same cells reconstituted with Fbw7α (*α*) were fixed and stained with LipidTOX Green neutral lipid stain. The mean fluorescence intensities -/+ SD across each experimental group are provided in the bar graph. **(B)** Fbw7 knockout (*KO*) HCT116 cells were transduced with control (*C*) or SREBP1 (*S1*) shRNA, fixed and stained with LipidTOX Green neutral lipid stain. The mean fluorescence intensities -/+ SD across each experimental group are provided in the bar graph. P-values lower than 0.05 were considered statistically significant. *P < 0.05, **P < 0.01, ***P < 0.001, and ****P < 0.0001. *NS*, not significant.

Taken together, our results thus far suggest that the inactivation of Fbw7α stabilizes nuclear SREBP1, leading to the enhanced expression of lipogenic genes, resulting in the accumulation of intracellular cholesterol and feedback inhibition of SREBP2 maturation. If this hypothesis is correct, Fbw7-deficient cells should accumulate high levels of neutral lipids, including cholesterol and triglycerides. To test this hypothesis, we used LipidTOX Green neutral lipid stain to monitor lipid accumulation in our three HCT116 cell models, i.e., wild-type, Fbw7-KO and Fbw7α cells. The wild-type cells displayed a moderate number of lipid droplets, which was greatly increased in the Fbw7-KO cells (**Figure 4A**). Interestingly, very few of the Fbw7α cells displayed any lipid droplets (**Figure 4A**), suggesting that Fbw7α is a strong inhibitor of neutral lipid accumulation. In agreement with the data in **Figures 3D, E**, the accumulation of lipid droplets in Fbw7-KO cells was significantly reduced following shRNA-mediated knockdown of SREBP1 (**Figure 4B**), suggesting that the stabilization of nuclear SREBP1 is important for the accumulation of neutral lipids following Fbw7 inactivation. Neutral lipids also accumulated in HepG2 cells following inactivation of Fbw7 in a manner dependent on SREBP1 (**Figure S5**).

### Expression of a cancer-associated loss-of-function version of Fbw7α stabilizes nuclear SREBP1 and attenuates the cleavage of SREBP2

**Figure 5:**
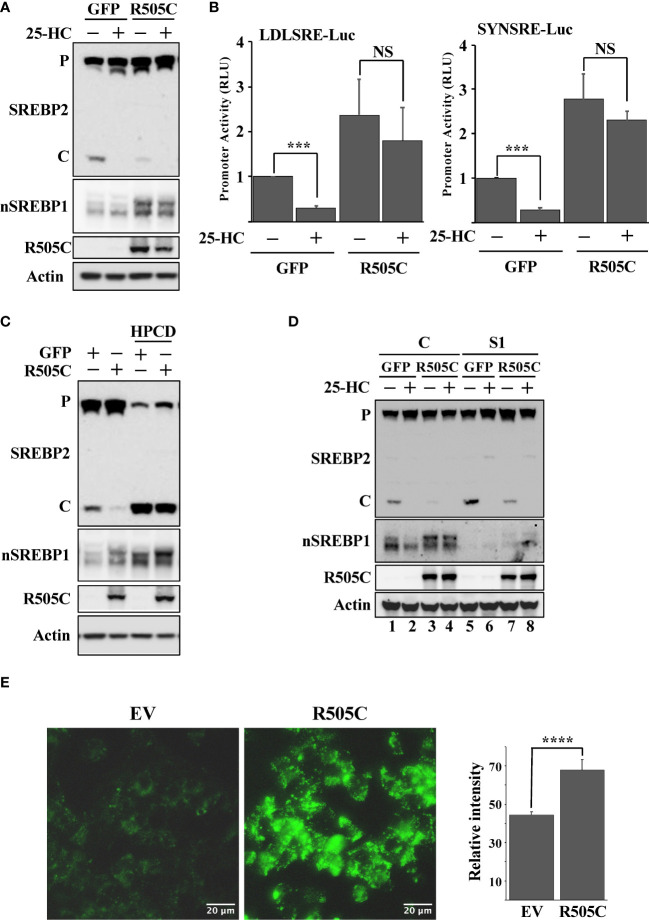
Expression of a cancer loss-of-function version of Fbw7α attenuates the maturation of SREBP2. **(A)** HepG2 cells were transduced with GFP or mutant Fbw7α (R505C) and treated without (-) or with (*+*) 25-HC for 6 hours. The levels of SREBP2, nuclear SREBP1 (*nSREBP1*), mutant Fbw7α (*R505C*) and β-actin in total lysates were determined by Western blotting. *P* and *C* denotes the precursor and cleaved forms of SREBP2, respectively. **(B)** HepG2 cells were transfected with luciferase promoter-reporter constructs containing the sterol-responsive portions of the human LDL receptor (*LDLRSRE-Luc*) or HMG-CoA synthase (*SYNSRE-Luc*) promoters together with empty vector or mutant Fbw7α (*R505C*). Where indicated, the transfected cells were treated with 25-HC for the last 3 hours of the experiment. At the end of the experiment, cells were lysed, and luciferase activity was measured. **(C)** HepG2 cells were transduced with GFP or Fbw7α (R505C) and left untreated (*-*) or treated (*+*) with HPCD for 2 hours. The levels of SREBP2, nuclear SREBP1 (*nSREBP1*), mutant Fbw7α (*R505C*) and β-actin in total lysates were determined by Western blotting. *P* and *C* denotes the precursor and cleaved forms of SREBP2, respectively. **(D)** HepG2 cells were transduced with either GFP or Fbw7α (R505C), followed by either control (*C*) or SREBP1 (*S1*) shRNA, and left untreated (*-*) or treated (*+*) with 25-HC for 6 hours. The levels of SREBP2, nuclear SREBP1 (*nSREBP1*), mutant Fbw7α (*R505C*), and β-actin in total lysates were determined by Western blotting. **(E)** HepG2 cells were transduced as in (*D*), fixed, and stained with LipidTOX Green neutral lipid stain. The mean fluorescence intensities -/+ SD across each experimental group are provided in the bar graph. P-values lower than 0.05 were considered statistically significant. *P < 0.05, **P < 0.01, ***P < 0.001, and ****P < 0.0001. *NS*, not significant.

Loss-of-function mutations in Fbw7 are frequently found in human cancers. We hypothesized that such mutations would result in the stabilization of nuclear SREBP1 and SREBP2, increased lipid metabolism, and the accumulation of intracellular lipids, thereby triggering the feedback mechanism controlling the maturation of SREBP2. In order to test this hypothesis, HepG2 cells were transduced with lentiviruses expressing either GFP (control) or a loss-of-function Fbw7α protein (R505C). The expression of this mutant resulted in the stabilization of nuclear SREBP1, a reduction in SREBP2 cleavage and a partial loss of 25-HC sensitivity (**Figure 5A**). The expression of mutant Fbw7α also enhanced the expression of SREBP-dependent promoter-reporter genes and made their expression insensitive to 25-HC (**Figure 5B**) as a result of the stabilization of nuclear SREBP1. As in the other loss-of-function models, the loss of SREBP2 cleavage in cells expressing mutant Fbw7α was reversed in response to HPCD-mediated cholesterol depletion (**Figure 5C**). In addition, inactivation of SREBP1 in the cells expressing mutant Fbw7α also restored SREBP2 cleavage and its responsiveness to 25-HC (**Figure 5D**, compare lanes 3 & 4 and 7 & 8). Taken together, our data suggest that loss-of-function Fbw7 mutations in cancer cells could result in the stabilization of pre-existing nuclear SREBP1 molecules, enhanced expression of SREBP target genes and the accumulation of intracellular lipids, thereby triggering the feedback mechanism controlling the activation of SREBP2. To test this hypothesis, we analyzed the accumulation of neutral lipids in cells expressing the non-functional Fbw7α mutant. Compared to control cells, cells expressing mutant Fbw7α accumulated significantly more lipid droplets labeled with the neutral lipid stain (**Figure 5E**).

### Expression of nuclear SREBP1a inhibits the cleavage of SREBP2

**Figure 6:**
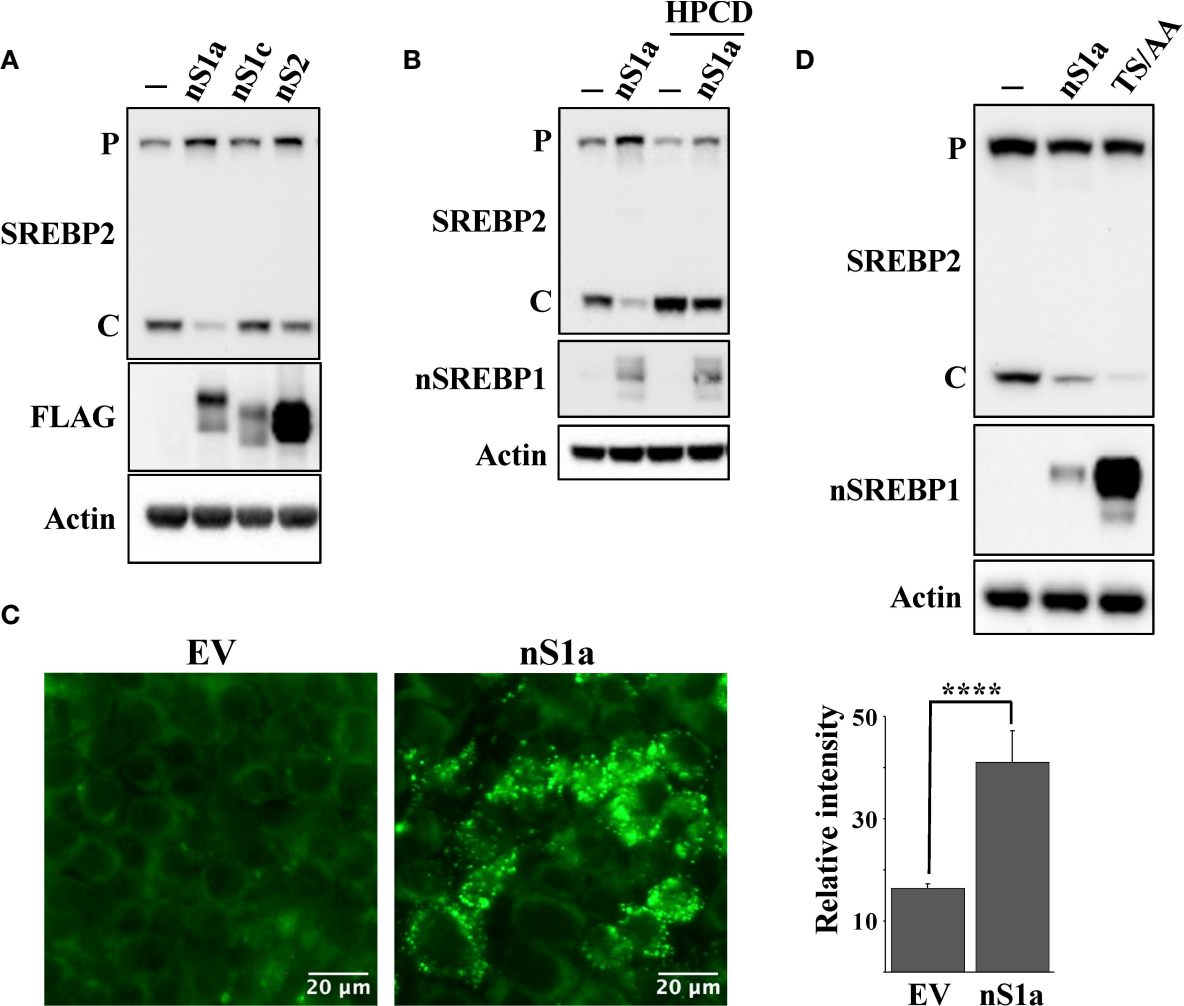
Nuclear SREBP1a attenuates the maturation of SREBP2. **(A)** HepG2 cells were transduced with GFP or the nuclear forms of SREBP1a (n*S1a*), SREBP1c (n*S1c*) or SREBP2 (n*S2*). The nuclear SREBP constructs contained a FLAG tag. The levels of endogenous SREBP2 and β-actin, and the transduced nuclear SREBPs (*FLAG*) in total lysates were determined by Western blotting. *P* and *C* denotes the precursor and cleaved forms of SREBP2, respectively. **(B)** HepG2 cells were transduced with GFP or the nuclear form of SREBP1a (n*S1a*) and were left untreated (-) or treated (*+*) with HPCD for 2 hours. The levels of SREBP2, nuclear SREBP1 (*nSREBP1*), and β-actin in total lysates were determined by Western blotting. *P* and C denotes the precursor and cleaved forms of SREBP2, respectively. (**C**) HepG2 cells were transduced with empty vector (*EV*) or nuclear SREBP1a (*nS1a*), fixed and stained with LipidTOX Green neutral lipid stain. The mean fluorescence intensities -/+ SD across each experimental group are provided in the bar graph. (**D**) HEK293 cells were transfected with empty vector, wild-type nuclear SREBP1a (*nS1a*) or a stabilized version of nuclear SREBP1a (*TS/AA*). The levels of endogenous SREBP2 and β-actin, and the transfected nuclear SREBP1a in total lysates were determined by Western blotting. *P* and *C* denotes the precursor and cleaved forms of SREBP2, respectively. Please note that the stabilized version of nuclear SREBP1a accumulates to much higher levels than the wild-type protein. P-values lower than 0.05 were considered statistically significant. *P < 0.05, **P < 0.01, ***P < 0.001, and ****P < 0.0001. *NS*, not significant.

The HepG2 (liver), HCT116 (colorectal) and MCF7 (breast) cell lines used in this work originate from human tumors and express SREBP1a, the strongest transcription factor in the SREBP family. Thus, since SREBP1a is able to transactivate both SREBP1 and SREBP2 target genes, we hypothesized that stabilization of nuclear SREBP1a was the main reason for the attenuated cleavage of SREBP2 in these cancer cell lines following Fbw7 inactivation. To test this assumption, HepG2 cells were transduced with lentiviruses expressing nuclear SREBP1a, SREBP1c, SREBP2 or the corresponding empty vector (control) and monitored the cleavage of endogenous SREBP2 by Western blotting. As seen in **Figure 6A**, expression of nuclear SREBP1a resulted in a significant decrease in the cleavage of endogenous SREBP2 while the effect of nuclear SREBP1c and SREBP2 were less obvious. The effect of SREBP1a expression on the cleavage of SREBP2 was reversed following HPCD treatment (**Figure 6B**), suggesting that the SREBP1a-dependent induction of cholesterol synthesis and/or uptake was an important factor in controlling the maturation of SREBP2. This interpretation was supported by our observation that HepG2 cells transduced with nuclear SREBP1a accumulated more neutral lipids compared to the same cells transduced with the empty vector (**Figure 6C**). If our overall hypothesis is correct, a stable form of nuclear SREBP1a should be more efficient in reducing the cleavage of SREBP2. Therefore, HEK293 cells were transfected with nuclear SREBP1a, either wild-type or a mutant form of the protein in which two phosphorylated threonine/serine residues within the Fbw7 phosphodegron were mutated to alanine residues (SREBP1a-TS/AA). As illustrated in **Figure 6D**, expression of wild-type SREBP1a resulted in a partial inhibition of SREBP2 cleavage, while expression of the stabilized mutant resulted in an almost complete loss of SREBP2 cleavage, suggesting that the stabilized form of SREBP1a was more efficient in stimulating cholesterol synthesis/uptake, resulting in a more robust feedback inhibition of SREBP2.

### Loss of Fbw7 results in activation of AKT

**Figure 7:**
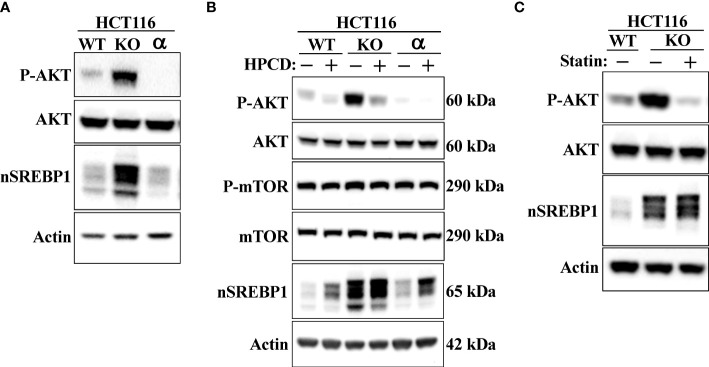
Loss of Fbw7α activates AKT. **(A)** HCT116 cells, either wild-type (*WT*), Fbw7 knockout (*KO*), or the same cells reconstituted with Fbw7α (*α*) were lysed and the levels of AKT, nuclear SREBP1 (*nSREBP1*), β-actin, and the phosphorylation of AKT on Ser473 (P-AKT) were determined by Western blotting. **(B)** HCT116 cells, either wild-type (*WT*), Fbw7 knockout (*KO*), or the same cells reconstituted with Fbw7α (*α*) were left untreated (-) or treated (*+*) with HPCD for 2 hours. The cells were lysed and the levels of AKT, mTOR, nuclear SREBP1 (*nSREBP1*), β-actin, and the phosphorylation of AKT on Ser473 (*P-AKT*) and mTOR on Ser2448 (*P-mTOR*) were determined by Western blotting. **(C)** Wild-type (WT) or Fbw7 knockout (*KO*) HCT116 cells were either left untreated (-) or treated (*+*) with fluvastatin (*Statin*) for 12 hours (2 μM), lysed and the levels of AKT, nuclear SREBP1 (*nSREBP1*), β-actin, and the phosphorylation of AKT on Ser473 (*P-AKT*) were determined by Western blotting.

Shao et al. recently demonstrated that cholesterol inhibits the autophagic degradation of receptor tyrosine kinases in human liver cancer cells by interfering with the function of GOLM1 (Golgi membrane protein 1) (58). As a result, the accumulation of excess intracellular cholesterol activated signaling from the stabilized receptors. If our hypothesis is correct, inactivation of Fbw7 should result in the accumulation of cholesterol and increased receptor tyrosine kinase signaling. Shao et al. demonstrated that HepG2 cells express very low levels of GOLM1 and therefore did not respond to cholesterol loading. Thus, we decided to use our HCT116 cell lines in our experiments. Because of the important role of the protein kinase AKT in the control of both metabolism and cell growth, we focused our attention on the activation of this kinase. As seen in **Figure 7A**, the phosphorylation/activation of AKT was higher in Fbw7-KO HCT116 cells compared to wild-type cells. Interestingly, the activation of AKT was very low in the Fbw7-KO cells reconstituted with Fbw7α, suggesting that the loss of Fbw7α results in activation of AKT. To determine if this effect was related to the accumulation of cholesterol in these cells, cells were treated with either HPCD, which rapidly removes cholesterol from the plasma membrane, or fluvastatin, a clinically relevant inhibitor of cholesterol synthesis. Treating Fbw7-KO cells with HPCD rapidly reduced the phosphorylation of AKT (**Figure 7B**). The same result was obtained following fluvastatin treatment of Fbw7-KO cells (**Figure 7C**). Interestingly, the phosphorylation of AKT was more sensitive to HPCD treatment in Fbw7-KO cells compared to wild-type cells. These data suggest that the inactivation of Fbw7α in HCT116 cells results in the accumulation of cholesterol and the activation of AKT. This notion was supported by our observation that the shRNA-mediated inactivation of Fbw7 in the breast cancer cell line MCF7 also resulted in the activation of AKT (**Figure S2**).

### SREBP1 is required for the activation of AKT in response to Fbw7 loss

**Figure 8:**
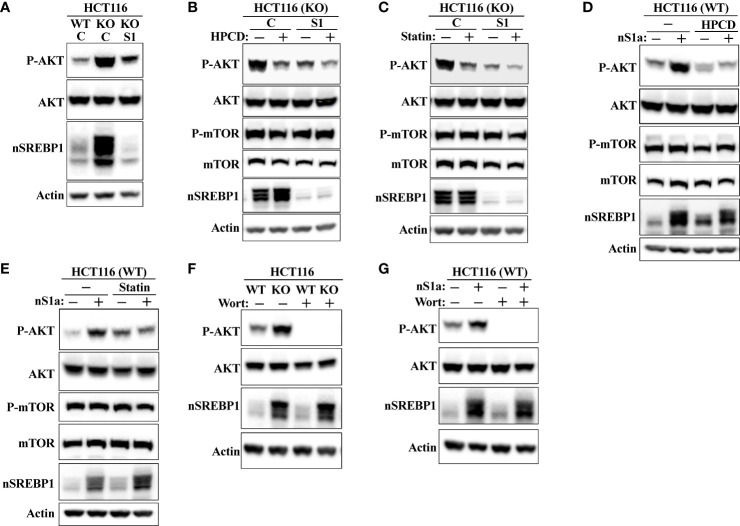
SREBP1 is required for the activation of AKT in response to Fbw7 loss. **(A)** Wild-type (*WT*) and Fbw7 knockout (*KO*) HCT116 cells were transduced with either control (*C*) or SREBP1 (*S1*) shRNA, lysed and the levels of AKT, nuclear SREBP1 (*nSREBP1*), β-actin, and the phosphorylation of AKT on Ser473 (*P-AKT*) were determined by Western blotting. **(B)** Fbw7 knockout (*KO*) HCT116 cells were transduced with either control (*C*) or SREBP1 (*S1*) shRNA and either left untreated (-) or treated (*+*) with HPCD for 2 hours, lysed and the levels of AKT, nuclear SREBP1 (*nSREBP1*), mTOR, β-actin, and the phosphorylation of AKT on Ser473 (*P-AKT*) and mTOR on Ser2448 (*P-mTOR*) were determined by Western blotting. **(C)** Wild-type and Fbw7 knockout (*KO*) HCT116 cells were transduced with either control (*C*) or SREBP1 (*S1*) shRNA and left untreated (-) or treated (*+*) with fluvastatin (*Statin*) for 12 hours (2 μM). The cells were lysed and the levels of AKT, mTOR, nuclear SREBP1 (*nSREBP1*), β-actin, and the phosphorylation of AKT on Ser473 (*P-AKT*) and mTOR on Ser2448 (*P-mTOR*) were determined by Western blotting. **(D)** Wild-type (*WT*) HCT116 cells were transduced with either GFP or nuclear SREBP1a (*nS1a*) and left untreated (-) or treated (+) with HPCD for 2 hours. The cells were lysed and the levels of AKT, mTOR, nuclear SREBP1 (*nSREBP1*), β-actin, and the phosphorylation of AKT on Ser473 (*P-AKT*) and mTOR on Ser2448 (*P-mTOR*) were determined by Western blotting. **(E)** Wild-type (*WT*) HCT116 cells were transduced with either GFP or nuclear SREBP1a (*nS1a*) and left untreated (-) or treated (*+*) with fluvastatin (*Statin*) for 12 hours (2 μM). The cells were lysed and the levels of AKT, mTOR, nuclear SREBP1 (*nSREBP1*), β-actin, and the phosphorylation of AKT on Ser473 (*P-AKT*) and mTOR on Ser2448 (*P-mTOR*) were determined by Western blotting. **(F)** Wild-type (*WT*) and Fbw7 knockout (*KO*) HCT116 cells were left untreated **(-)** or treated (*+*) with wortmannin (*Wort*, 200 nM) for 1 hour. The cells were lysed and the levels of AKT, nuclear SREBP1 (*nSREBP1*), β-actin, and the phosphorylation of AKT on Ser473 (*P-AKT*) were determined by Western blotting. **(G)** Wild-type (*WT*) HCT116 cells were transduced with either GFP or nuclear SREBP1a (*nS1a*) and treated as in (*F*). The cells were lysed and the levels of AKT, nuclear SREBP1 (*nSREBP1*), β-actin, and the phosphorylation of AKT on Ser473 (*P-AKT*) were determined by Western blotting.

shRNA-mediated inactivation of SREBP1 in Fbw7-KO cells reduced the phosphorylation of AKT (**Figure 8A**), suggesting that the stabilization of nuclear SREBP1 in these cells could be important for the activation of AKT. This possibility was supported by our observation that the inactivation of SREBP1 in Fbw7-KO cells attenuated the effects of HPCD (**Figure 8B**) and fluvastatin (**Figure 8C**) on the phosphorylation of AKT, i.e., inactivation of SREBP1 reduced the phosphorylation of AKT and no further reduction was observed following either treatment. In addition, the phosphorylation of AKT was reduced in response to SREBP1 knockdown in MCF7 cells (**Figure S6**). Importantly, inactivation of SREBP1 also prevented the activation of AKT in Fbw7-deficient MCF7 cells (**Figure S3**). Forced expression of nuclear SREBP1a in wild-type HCT116 cells increased the phosphorylation of AKT (**Figures 8D, E**). Importantly, the SREBP1a-dependent activation of AKT was lost in cells treated with either HPCD (**Figure 8D**) or fluvastatin (**Figure 8E**), suggesting that the ability of nuclear SREBP1a to activate AKT was dependent on the intracellular accumulation of cholesterol.

AKT is a major target of PI3K downstream of a number of receptor tyrosine kinases. To determine if the increased activation of AKT in response to Fbw7 loss was dependent on PI3K, wild-type and Fbw7-KO cells were treated in the absence or presence of the PI3K inhibitor wortmannin. As seen in **Figure 8F**, the phosphorylation of AKT was lost in both cell lines in response to wortmannin treatment. The same result was obtained in wild-type cells transduced with viruses expressing nuclear SREBP1a (**Figure 8G**). Taken together, our data suggest that the stabilization of nuclear SREBP1a in response to Fbw7 loss results in the intracellular accumulation of cholesterol, which in turn leads to the activation of the PI3K-AKT signaling axis. AKT is both a substrate and activator of mTOR (34, 38, 39), and it has been suggested that mTOR is a substrate of Fbw7 (59). Thus, the enhanced phosphorylation of AKT in the Fbw7-KO cells could be the result of the accumulation of mTOR in these cells. However, we were unable to detect any difference in the levels or activation of mTOR between wild-type and Fbw7-KO HCT116 cells (**Figure S7**).

**Figure 9:**
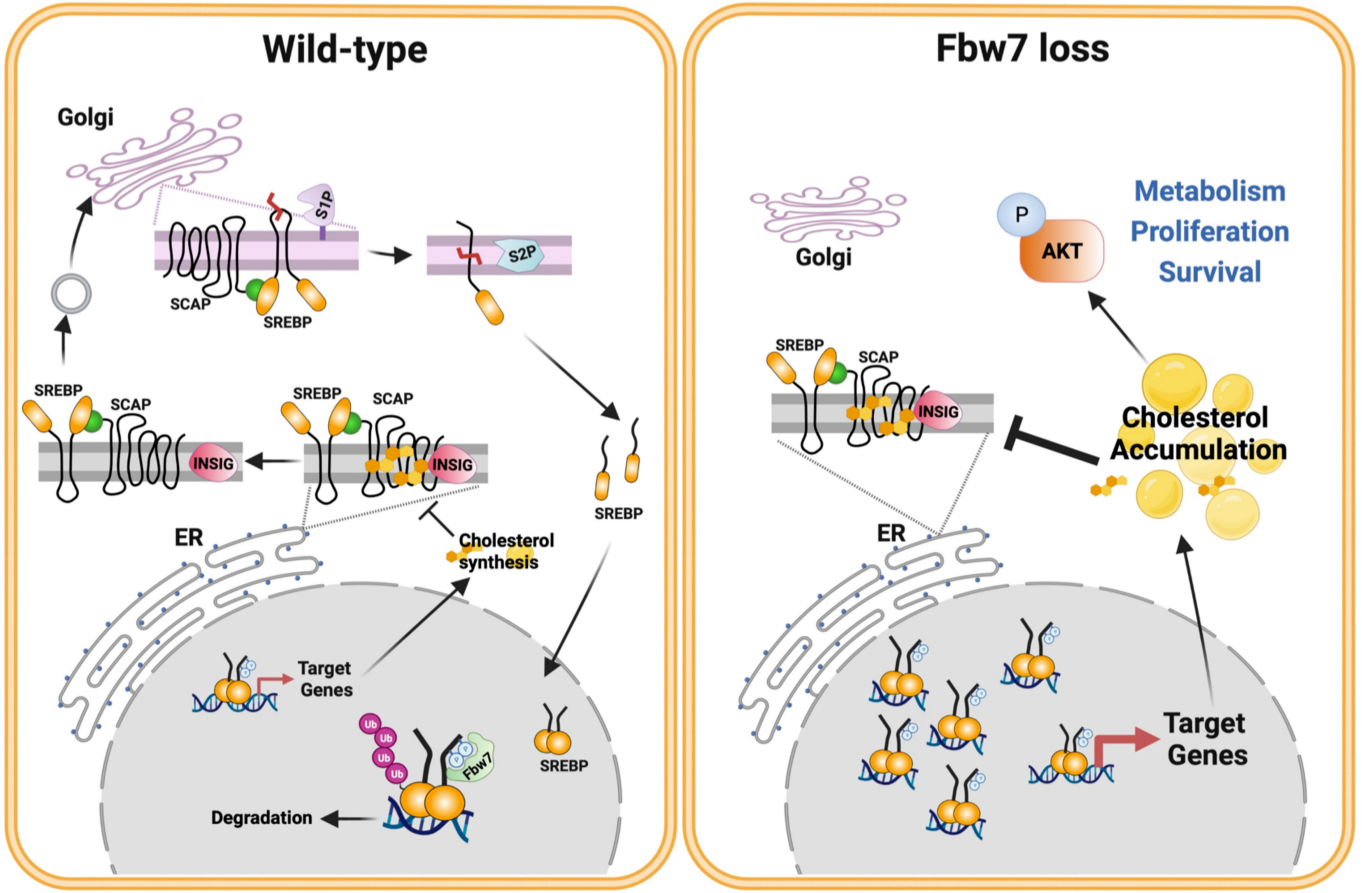
Fbw7α loss in cancer cells rewires cholesterol metabolism resulting in activation of PI3K-AKT signaling. Based on the results of this investigation, we propose the following model. The activation of the SREBP family of transcription factors is controlled by the interaction between INSIG and SCAP and the SCAP-dependent transport of precursor SREBP molecules from the ER to the Golgi. Once in the Golgi, the precursor proteins are proteolytically cleaved by two separate proteases, site-1 protease (S1P) and S2P, thereby releasing the active transcription factors. The release of the SCAP-SREBP complex from the ER is inhibited by cholesterol. Under normal conditions (*left*), nuclear SREBP molecules are phosphorylated by GSK3 and targeted for ubiquitination and degradation by the tumor suppressor and E3 ligase Fbw7α. This process ensures normal cholesterol homeostasis. However, this process is disrupted in response to loss of Fbw7α protein and/or function (*right*). As a result, the levels of nuclear SREBP molecules accumulate and the expression of target genes is increased, including genes involved in cholesterol and fatty acid synthesis/metabolism. Cancer cells express SREBP1a, the most potent transcriptional activator of the SREBP family, capable of activating both SREBP1 and SREBP2 target genes. As a result, loss of Fbw7α in cancer cells results in a significant increase in both cholesterol and fatty acid synthesis. We propose that this provides cancer cells with an ample supply of lipids to support their rapid proliferation. Our data suggest that the accumulation of cholesterol in response to SREBP1a stabilization triggers the negative feedback loop restricting SREBP2 maturation, which could prevent the build up of toxic levels of cholesterol and/or cholesterol metabolites in cancer cells. Our data also suggest that the accumulation of cholesterol in response to Fbw7α loss activates AKT, a major positive regulator of cancer cell metabolism, proliferation and survival. Model designed by MTBA and created with BioRender.com.

The current study confirms our previous findings that the loss of Fbw7 results in the stabilization of nuclear SREBP1. We also demonstrate that the stabilization of nuclear SREBP1a results in the sterol-insensitive activation of lipid synthesis, something that could support enhanced cell growth. We also find that the stabilization of nuclear SREBP1a, and the resulting accumulation of intracellular cholesterol, triggers the well-established negative feedback loop of SREBP2 cleavage/activation. In theory, this could help reduce the accumulation of toxic levels of cholesterol or cholesterol metabolites. We also report that the loss of Fbw7 in cancer cells results in the enhanced phosphorylation/activation of AKT in a manner dependent on cholesterol and SREBP1. AKT is a major signaling hub downstream of receptor tyrosine kinase activation and controls cell metabolism, growth and survival. Thus, we propose that the loss of the tumor suppressor Fbw7 in cancer cells could provide these cells with an advantage as a result of SREBP1a stabilization and cholesterol-mediated activation of AKT (**Figure 9**, model).

## Discussion

Nuclear SREBP1a, SREBP1c and SREBP2 are targeted for ubiquitin-dependent degradation by SCF^Fbw7^, a cullin-1-dependent ubiquitin ligase. While trying to identify novel ubiquitin ligases regulating the SREBP pathway, we discovered that inactivation of cullin 1, a component of many SCF-type ubiquitin ligases, blocked the maturation of SREBP2. We were subsequently able to show that this was caused by the inactivation of Fbw7, the ubiquitin ligase that targets nuclear SREBP1 and SREBP2 for degradation. Inactivation of cullin 1 or Fbw7 induced the expression of SREBP target promoter-reporter genes and rendered them insensitive to 25-hydroxycholesterol, a well-established inhibitor of the SREBP pathway. The same was true for endogenous target genes in Fbw7-KO HCT116 cells. Importantly, the same effects were seen in cells expressing a mutant version of Fbw7 found in many human cancers. At the same time, the Fbw7-deficient cells accumulated higher levels of neutral lipids compared to their parental control cells. Thus, inactivation of Fbw7 stabilizes nuclear SREBP1 and results in the activation of lipogenic target genes despite the accumulation of excess lipids. It also blocks the cleavage of SREBP2, possibly a consequence of the accumulation of cholesterol in the ER. This hypothesis was supported by our observation that the cleavage of SREBP2 in Fbw7-deficient cells could be restored by treating the cells with HPCD, which rapidly depletes cells of cholesterol. Interestingly, inactivation of SREBP1 also restored the cleavage of SREBP2 and the sterol-dependent expression of both SREBP-regulated promoter-reporter genes and endogenous target genes. In addition, the forced expression of nuclear SREBP1a inhibited the cleavage of SREBP2, and this effect was more pronounced in cells expression a mutant version of SREBP1a that is unable to interact with Fbw7. Fbw7 exists in three isoforms, Fbw7α, Fbw7β, and Fbw7γ, and only one of these, Fbw7α, targets nuclear SREBP molecules. Notably, reconstituting Fbw7-deficient cells with the α isoform was sufficient to restore the cleavage of SREBP2 and the sterol-regulated expression of target genes. Taken together, our results suggest that the inactivation of Fbw7α in human cancer cells results in the stabilization of nuclear SREBP1a, thereby activating the expression of target genes involved in fatty acid and cholesterol synthesis. The enhanced expression of these genes could be important to support the increased demand for lipids in rapidly growing cells. At the same time, the accumulation of cholesterol in these cells blocks the cleavage and activation of SREBP2. Theoretically, this could protect cells from accumulating toxic levels of cholesterol and/or cholesterol metabolites. The activation of a cholesterol-dependent negative feedback loop could also help explain the relatively modest accumulation of lipids observed in liver-specific Fbw7-KO mice (60). However, this possibility will have to be explored further, including studies aimed at determining the lipid profiles of Fbw7-deficient cells. In addition, we only looked at cancer cell lines in the current study, all of which express SREBP1a, a strong activator of both fatty acid and cholesterol synthesis. Thus, it will be important to determine the effect of Fbw7 inactivation in non-cancer cells. Although the general consensus is that most human tissues express low levels of SREBP1a, this notion has been challenged in studies using primary human hepatocytes (61). We are currently exploring the functional consequences of Fbw7 inactivation in human iPSC-derived hepatocytes.

Cholesterol is a highly hydrophobic molecule, confined primarily to lipid membranes and lipid droplets, including lipoprotein particles. However, it is not simply an inert component of these structures. In fact, cholesterol plays an important role in controlling membrane structure and function. It is synthesized within the ER, but the level of cholesterol is usually very low in this membrane, which makes it a very good sensor of intracellular cholesterol levels (12, 62). Instead, newly synthesized cholesterol is rapidly transported from the ER to other intracellular membranes, with a large portion ending up within the plasma membrane. Cholesterol has been shown to influence the structure and function of the plasma membrane and aid in the formation of subdomains within the membrane, such as lipid rafts, and regulate endo- and exocytosis (63–66). Signals originating from plasma membrane rafts affect multiple cellular processes, with many being intimately linked to cancer (64, 65, 67). In addition, cholesterol plays important roles in Hedgehog signaling and many proteins in this pathway share a sterol sensing domain found in components of the SREBP pathway (68–71). Work in Richard Anderson’s laboratory demonstrated that cholesterol is directly involved in intracellular signaling by regulating the MAPK signaling pathway (72, 73).

In a recent study, Shao et al. demonstrated that cholesterol inhibits the degradation of receptor tyrosine kinases in human liver cancer cells (58). As a result, the accumulation of excess intracellular cholesterol activated these receptors and their downstream targets. Importantly, the authors showed that lowering cholesterol by statins (lovastatin) improved the efficacy of multiple tyrosine kinase inhibitors in *in vivo* tumor models. In the current manuscript, we found that AKT is activated in Fbw7-deficient HCT116 cells compared to their parental controls. Importantly, the activation of AKT was reversed if these cells were treated with HPCD or fluvastatin, both of which reduce intracellular cholesterol levels. In addition, inactivation of endogenous SREBP1 in Fbw7-deficient cells reduced the activation of AKT in these cells. We also found that the forced expression of nuclear SREBP1a in wild-type cells resulted in AKT activation. Importantly, we found that the activation of AKT in response to SREBP1a expression was lost if cells were treated with HPCD or fluvastatin, demonstrating that the ability of SREBP1a to induce AKT phosphorylation is dependent on the accumulation of cholesterol. In addition, the induction of AKT downstream of Fbw7 loss and SREBP1a activation was dependent on PI3K, a well-established proto-oncogene. Thus, our work confirms the observations made by Shao et al. suggesting that cholesterol is able to activate the PI3K-AKT signaling axis. Our work also extends their original observations to colorectal and breast cancer cells and demonstrates that these processes can be regulated by the Fbw7 tumor suppressor and components of the SREBP pathway, primarily SREBP1a. However, it is unclear if the mechanisms reported here are the same as those reported by Shao et al. We focused our studies on the activation AKT, while Shao et al. focused on mTOR. Although we did not monitor mTOR activity in all our experiments, we did not observe a correlation between the phosphorylation/activation of AKT and mTOR in the experiments where the activation of both proteins were monitored. However, our results do not exclude the activation of mTOR in our cell models. It is possible that mTOR is activated through slightly different mechanisms in the cell lines used, but it is more likely that the timing of mTOR activation downstream of cholesterol loading/depletion diverges between cell lines. In addition, our experiments were focused on the reduction of intracellular cholesterol levels, while Shao et al. mainly focused on the addition of exogenous cholesterol to cholesterol-depleted cells. Regardless, both studies support the notion that the accumulation of excess cholesterol results in activation of the PI3K-AKT-mTOR signaling pathway, suggesting that reducing intracellular cholesterol levels could be an attractive therapeutic target in certain human cancers. However, further mechanistic work is required to establish how cholesterol regulates the activation of AKT. For example, it will be important to determine if this is accomplished at the level of specific plasma membrane receptors. In addition, Shao et al. demonstrated the important role of the autophagy-related protein GOLM1 in liver cancer cells. Thus, it will be important to clarify if this protein is also involved in the activation of AKT in our cell models. It will also be important to explore the functional consequences of AKT activation in response to cholesterol accumulation.

It has been reported that Fbw7 targets mTOR for degradation (59). Thus, since AKT is activated through mTORC2-mediated phosphorylation it was possible that the accumulation of mTOR in response to Fbw7 loss could explain the increased phosphorylation of AKT in our Fbw7 knockout and knockdown cells. However, we were unable to detect any significant difference in the expression of mTOR protein and/or activation in our Fbw7 knockout or knockdown cells, suggesting that it is unlikely that this could explain our results. One possible explanation is that Fbw7 is not a major regulator of mTOR stability in our cell models and/or under the experimental conditions used in this study. Regardless, it is well-established that mTORC2 contributes to the phosphorylation and activation of AKT and the involvement of mTORC2 in the cholesterol-dependent activation of AKT should be explored in future work.

In summary, our data show that inactivation of the E3 ubiquitin ligase Fbw7 in cancer cells results in the stabilization of nuclear/active SREBP1a, resulting in the activation of lipid synthesis. This in turn results in the inactivation of the cleavage and activation of SREBP2 by triggering the well-established negative feedback loop between intracellular cholesterol levels and SREBP2 activation. Furthermore, our data suggest that the accumulation of excess cholesterol downstream of SREBP1a stabilization activates the protein kinase AKT downstream of PI3K. AKT, through its activation of mTOR and inactivation of GSK3, is an important positive regulator of the SREBP pathway. Our data suggest that the SREBP pathway is not only a target of AKT-dependent signaling but could also be a regulator of this important signaling hub by its ability to control intracellular cholesterol metabolism, at least in cancer cells. Thus, loss of the Fbw7 tumor suppressor and the stabilization of nuclear SREBP1a could provide cancer cells with multiple advantages. Firstly, it ensures sustained *de novo* lipid synthesis to support rapid proliferation. Secondly, the rewiring of cholesterol metabolism downstream of SREBP1a stabilization results in activation of AKT, an important regulator of cell metabolism, proliferation, and survival. Future studies should explore if the cholesterol-dependent regulation of AKT-mTOR signaling contributes to cancer initiation/progression. This may be especially important in cancers overrepresented in patients with metabolic disease, such as endometrial, liver, pancreatic and colorectal cancer.

## Data availability statement

The raw data supporting the conclusions of this article will be made available by the authors, without undue reservation.

## Author contributions

MB-A and JE conceptualized the study. JE and MB-A conducted experiments and performed data analysis, and manuscript writing. AA performed qPCR assays and experiments in MCF7 cells. JE secured funding, designed the research, and performed data analysis and manuscript writing. All authors contributed to article writing and approved the submitted version of the manuscript.

## Funding

This study was supported by the Qatar National Research Fund (NPRP13S-0127-200178). Open Access funding provided by the Qatar National Library.

## Acknowledgments

The authors thank Ayman Al Haj Zen and Lutfiye Yildiz Ozer for assistance with microscopy and image analysis, members of the Ericsson laboratory for their feedback, and the College of Health and Life Sciences at HBKU for intramural support.

## Conflict of interest

The authors declare that the research was conducted in the absence of any commercial or financial relationships that could be construed as a potential conflict of interest.

## Publisher’s note

All claims expressed in this article are solely those of the authors and do not necessarily represent those of their affiliated organizations, or those of the publisher, the editors and the reviewers. Any product that may be evaluated in this article, or claim that may be made by its manufacturer, is not guaranteed or endorsed by the publisher.
