# Supplementary material for: Loss of the Fbw7 tumor suppressor rewires cholesterol metabolism in cancer cells leading to activation of the PI3K-AKT signalling axis
Source: Front Oncol. 2022 Sep 13;12:990672. doi: 10.3389/fonc.2022.990672 (PMC9513553; doi:10.3389/fonc.2022.990672)
Supplement: Supplementary file 1 [file DataSheet_1.pdf]

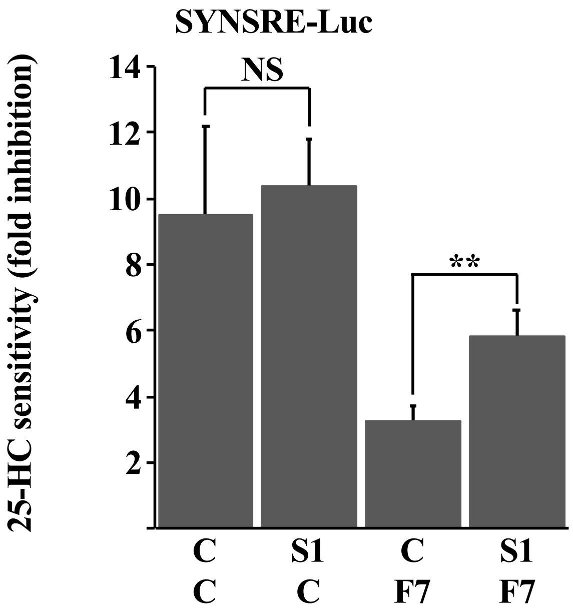

Figure S1. SREBP1 regulates the sterol-sensitivity of the HMG-CoA synthase promoter in Fbw7-deficient cells. HepG2 cells were transfected with the HMG-CoA synthase promoter-reporter gene (SYNSRE-Luc) together with control (*C*), Fbw7 (*F7*) or SREBP1 (*S1*) shRNA as indicated. Forty-eight hours following transfection, the cells were left untreated or treated with 25-HC for 6 hours. Subsequently, the cells were lysed, and luciferase activity was measured. The results are displayed as the fold inhibition in response to 25-HC. The sterol sensitivity of the promoter-reporter in Fbw7-deficient cells was partially restored following SREBP1 inactivation. P-values lower than 0.05 were considered statistically significant. *P < 0 .05, **P < 0 .01, ***P < 0 .001, and ****P < 0.0001. *NS*, not significant. Related to Fig. 2.

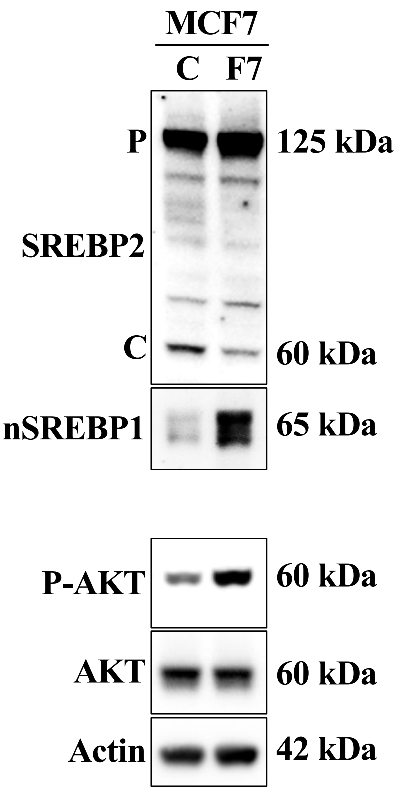

Figure S2. Inactivation of Fbw7 in MCF7 cells attenuates SREBP2 maturation and activates AKT. MCF7 cells were transduced with control (*C*) or Fbw7 (*F7*) shRNA as indicated. Forty-eight hours following transduction, the cells were lysed and the levels of SREBP2, nuclear SREBP1 (*nSREBP1*), AKT, β-actin and the phosphorylation of AKT on S473 (*P-AKT*) in total lysates were determined by Western blotting. *P* and *C* denotes the precursor and cleaved forms of SREBP2, respectively. The cleavage of SREBP2 was relatively low in MCF7 cells but was further reduced in response to Fbw7 inactivation. The phosphorylation of AKT was increased in response to Fbw7 inactivation.

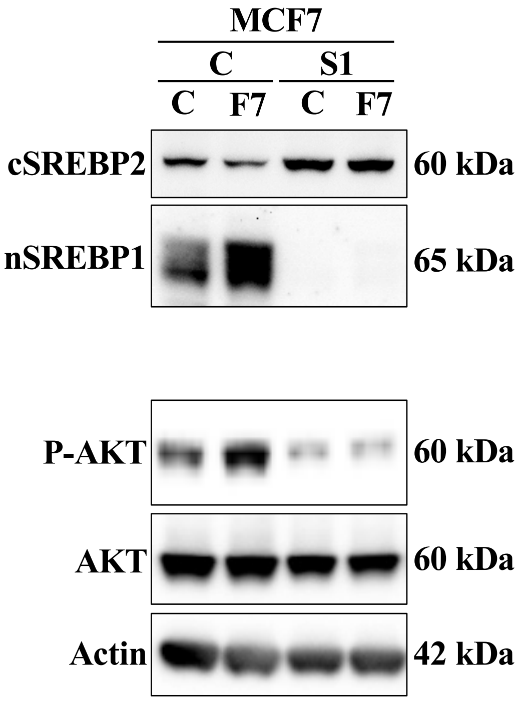

Figure S3. The Fbw7-dependent regulation of SREBP2 maturation and AKT phosphorylation in MCF7 cells require SREBP1. MCF7 cells were transduced with control (*C*) or SREBP1 (*S1*) shRNA followed by either control (*C*) or Fbw7 (*F7*) shRNA. The levels of cleaved SREBP2 (*cSREBP2*), nuclear SREBP1 (nSREBP1), AKT, β-actin and the phosphorylation of AKT on S473 (P-AKT) in total lysates were determined by Western blotting. The cleavage of SREBP2 was relatively low in MCF7 cells but was further reduced in response to Fbw7 inactivation. Inactivation of SREBP1 enhanced the maturation of SREBP2, especially in the Fbw7-deficient cells. Inactivation of SREBP1 reduces the phosphorylation of AKT and cancels out the induction observed in response to Fbw7 knockdown.

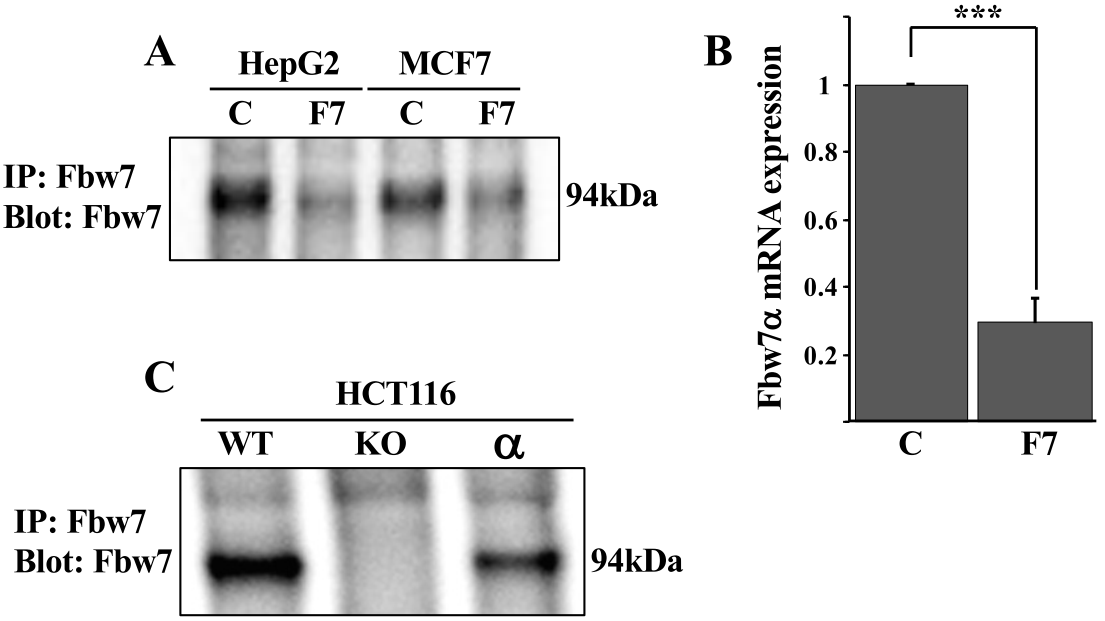

Figure S4. Confirmation of Fbw7 knockdown efficiency in HepG2 and MCF7 cells and the Fbw7 status of HCT116 cells. (**A**) HepG2 and MCF7 cells were transduced with control (*C*) or Fbw7 (*F7*) shRNA as indicated. Forty-eight hours following transduction, the cells were lysed and Fbw7 was immunoprecipitated followed by SDS-PAGE and Western blotting. (**B**) HepG2 cells were transduced as in (*A*) followed by qPCR to determine the expression of Fbw7α. The expression of Fbw7α in control cells was set to 1. (**C**) Fbw7 was immunoprecipitated from total cell lysates from HCT116 cells, either wild-type (*WT*), Fbw7 knockout (*KO*) or the same cells reconstituted with Fbw7α (*α*) and resolved by SDS-PAGE and analyzed by Western blotting. P-values lower than 0.05 were considered statistically significant. *P < 0 .05, **P < 0 .01, ***P < 0 .001, and ****P < 0.0001. *NS*, not significant. Related to Fig. 3.

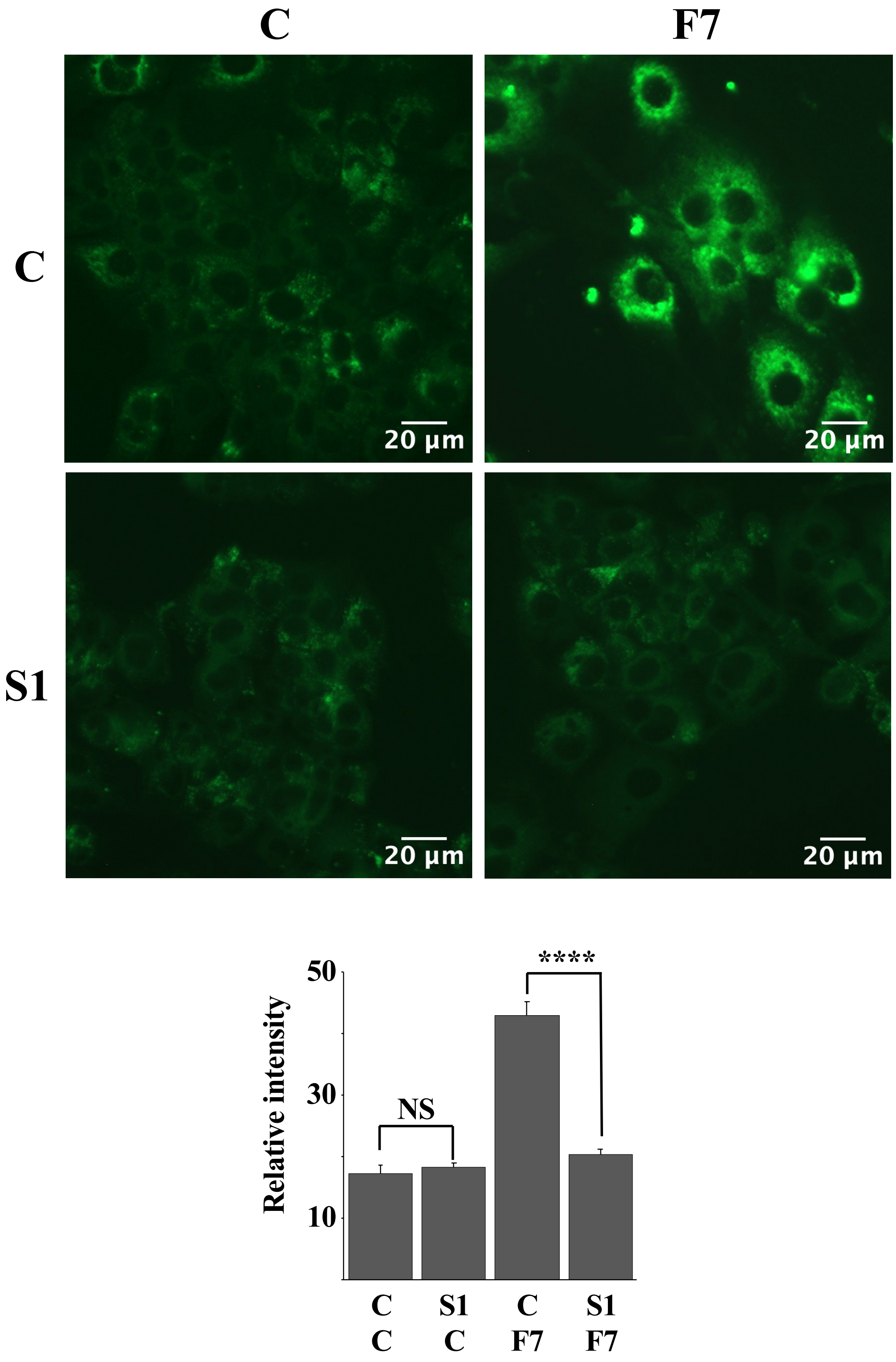

Figure S5. Fbw7 regulates the accumulation of neutral lipids in HepG2 cells. HepG2 were transduced with control (*C*, upper 2 panels) or SREBP1 (*S1*, lower 2 panels) shRNA, followed by control (*C*, left) or Fbw7 (*F7*, right) shRNA. Forty-eight hours following the second transduction, cells were fixed and stained with LipidTOX Green neutral lipid stain. All images were captured using the same microscope settings. The mean fluorescence intensities -/+ SD across each experimental group are provided in the bar graph. P-values lower than 0.05 were considered statistically significant. *P < 0 .05, **P < 0 .01, ***P < 0 .001, and ****P < 0.0001. NS, not significant. Related to Fig. 4.

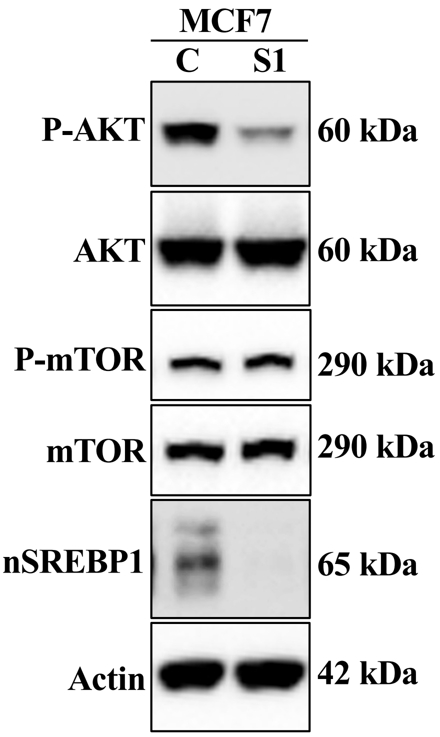

Figure S6. SREBP1 regulates AKT phosphorylation in MCF7 cells. (**A**) MCF7 cells were transduced with control (C) or SREBP1 (*S1*) shRNA and the levels of nuclear SREBP1 (*nSREBP1*), AKT, mTOR, β-actin, and the phosphorylation of AKT on S473 (*P-AKT*) and mTOR on Ser2448 (*P-mTOR*) in total lysates were determined by Western blotting.

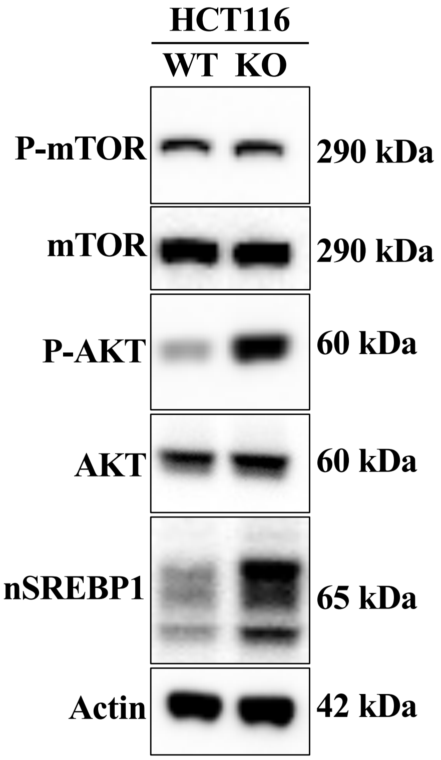

Figure S7. mTOR does not accumulate in Fbw7 knockout HCT116 cells. HCT116 cells, either wild-type (*WT*) or Fbw7 knockout (*KO*), were lysed and the levels of mTOR, nuclear SREBP1 (*nSREBP1*), AKT, β-actin and the phosphorylation of AKT on S473 (*P-AKT*) and mTOR on Ser2448 (*P-mTOR*) in total lysates were determined by Western blotting. Related to Figs. 7 and 8.
